# Chest X-ray sensitivity and lung cancer outcomes: a retrospective observational study

**DOI:** 10.3399/BJGP.2020.1099

**Published:** 2021-07-27

**Authors:** Stephen H Bradley, Bobby SK Bhartia, Matthew EJ Callister, William T Hamilton, Nathaniel Luke Fielding Hatton, Martyn PT Kennedy, Luke TA Mounce, Bethany Shinkins, Pete Wheatstone, Richard D Neal

**Affiliations:** Leeds Institute of Health Sciences, University of Leeds, Leeds.; Leeds Teaching Hospitals NHS Trust, Leeds.; Leeds Teaching Hospitals NHS Trust, Leeds.; College of Medicine and Health, University of Exeter, Exeter.; Leeds Teaching Hospitals NHS Trust, Leeds.; Leeds Teaching Hospitals NHS Trust, Leeds.; College of Medicine and Health, University of Exeter, Exeter.; Test Evaluation Group, Leeds Institute of Health Sciences, University of Leeds, Leeds.; CanTest Collaborative, c/o Academic Unit of Primary Care, University of Leeds, Leeds.; Academic Unit of Primary Care, University of Leeds, Leeds.

**Keywords:** chest X-ray, early diagnosis, general practice, lung cancer, radiograph, test accuracy

## Abstract

**Background:**

Chest X-ray (CXR) is the first-line investigation for lung cancer in many healthcare systems. An understanding of the consequences of false-negative CXRs on time to diagnosis, stage, and survival is limited.

**Aim:**

To determine the sensitivity of CXR for lung cancer and to compare stage at diagnosis, time to diagnosis, and survival between those with CXR that detected, or did not detect, lung cancer.

**Design and setting:**

Retrospective observational study using routinely collected healthcare data.

**Method:**

All patients diagnosed with lung cancer in Leeds Teaching Hospitals NHS Trust during 2008–2015 who had a GP-requested CXR in the year before diagnosis were categorised based on the result of the earliest CXR performed in that period. The sensitivity of CXR was calculated and analyses were performed with respect to time to diagnosis, survival, and stage at diagnosis.

**Results:**

CXR was negative for 17.7% of patients (*n* = 376/2129). Median time from initial CXR to diagnosis was 43 days for those with a positive CXR and 204 days for those with a negative CXR. Of those with a positive CXR, 29.8% (95% confidence interval [CI] = 27.9% to 31.8%) were diagnosed at stage I or II, compared with 33.5% (95% CI = 28.8% to 38.6%) with a negative CXR.

**Conclusion:**

GPs should consider lung cancer in patients with persistent symptoms even when CXR is negative. Despite longer duration to diagnosis for those with false-negative CXRs, there was no evidence of an adverse impact on stage at diagnosis or survival; however, this comparison is likely to be affected by confounding variables.

## INTRODUCTION

Lung cancer is the world’s leading cause of cancer mortality.^1^ Since those who are diagnosed at an earlier stage of disease have improved outcomes, there has been a heavy emphasis in cancer policy on streamlining diagnosis.^2^ For example, England’s NHS aims to achieve diagnosis at stage I or II in three-quarters of all patients who have cancer by 2028.^3^ Given the central role of chest X-ray (CXR) in lung cancer diagnosis in countries such as the UK, it is important to understand the ability of CXRs to detect lung cancer and the possible adverse implications on outcomes when lung cancer is not detected.^4^ There is currently insufficient high-quality evidence to address these questions.

Studies with a low risk of bias that were identified in a systematic review,^5^ along with a subsequently published study,^6^ have estimated that CXR does not identify lung cancer in approximately 20%–25% of cases. The pooled number of individuals with lung cancer from these studies was relatively small (*n* = 494), and definitions of positive and negative results were not entirely consistent between studies. Evidence regarding the consequences of false-negative CXR results in terms of time to diagnosis, stage at diagnosis, and survival is even more limited. A case series and two diagnostic audits suggest that those with false-negative CXRs may experience a greater time to diagnosis.^7^^–^^9^ A retrospective review of 28 patients found no adverse association between survival and ‘missed’ lung cancer on CXR.^10^

Using routinely collected data, this study aimed to calculate the sensitivity of GP-initiated CXR for lung cancer in the year before diagnosis and to compare time to diagnosis from CXR, stage at diagnosis, and survival between patients who had positive and negative CXR results for lung cancer in the year before diagnosis.

## METHOD

Leeds Teaching Hospitals NHS Trust (LTHT) is a regional centre for lung cancer diagnosis and treatment, serving a population of approximately 750 000.^11^ LTHT’s lung cancer database is a comprehensive record of multidisciplinary team-confirmed lung cancer diagnoses, which has previously been described.^12^ From this, a database was created containing de-identified data on all patients diagnosed with a primary lung cancer between 1 January 2008 and 31 December 2015 at LTHT. This included lung cancer cases that conformed to the International Classification of Diseases diagnostic code C34;^13^ therefore other intrathoracic malignancies such as mesothelioma were excluded. Patients who did not have a CXR requested by their GP in the year before they were diagnosed with lung cancer were excluded. All radiology reports for GP-requested CXRs in the year before diagnosis were coded according to criteria adapted from a national audit.^14^ The CXR report codes were as follows:

1. Suspicion of lung cancer identified/urgent investigation indicated.
2. Abnormality identified/non-urgent investigation indicated, including diagnoses of pneumonia or consolidation even if repeat imaging was not explicitly suggested.
3. Abnormality identified but no further investigation/assessment indicated.
4. Normal CXR: no abnormalities identified.

**Table:** How this fits in

An understanding of the accuracy of chest X-rays (CXRs) for diagnosing lung cancer in people with symptoms is limited, and little is known about adverse consequences when the investigation does not identify cancer. Analysing CXR results for >2000 patients, this study demonstrated that the sensitivity of CXRs was 82.3%. Aside from a longer time to diagnosis, no adverse consequences in terms of survival or stage of disease were observed for patients who had a CXR that did not detect lung cancer; however, these results could be explained by confounding factors. GPs should be aware that CXRs may initially miss lung cancer in around a fifth of cases and should consider further investigation if symptoms persist.

Codes 1 and 2 were considered to be ‘positive’ results, while codes 3 and 4 were ‘negative’. A sample of 100 CXR reports were independently categorised by two researchers. This yielded Cohen’s κ scores of 0.80 and 0.92 on comparing agreement across all four codes (1–4) and into the positive (1–2) versus negative (3–4) categories, respectively. Coding was subsequently performed by one researcher with advice obtained from another researcher on the categorisation of results that were ambiguous.

Patients were categorised according to the code of the earliest GP-requested CXR in the year before diagnosis (initial CXR) into four groups. This period was chosen because it is likely that cancer would be present during this interval before diagnosis.^15^ The date of diagnosis was the date of biopsy confirmation or of the multidisciplinary team meeting’s decision to accept a radiological diagnosis, which occurs in instances when biopsy is not obtained, for example, if a patient is too ill to tolerate the procedure.

### Statistical analysis

Sensitivity was calculated as the proportion of patients who had an initial CXR coded as either 1 or 2. Pearson’s χ^2^ test was used to determine if a statistically significant association was present between early-stage (I and II) and late-stage (III and IV) disease and positive and negative CXR results.

Kaplan–Meier survival curves were calculated to compare ‘true-positive’ and ‘false-negative’ groups in terms of survival from initial CXR and duration from initial CXR to lung cancer diagnosis. The log rank test was used to test the null hypothesis that there was no difference in survival between these two groups. A Cox proportional hazards model was fitted to allow adjustment for age, sex, deprivation, performance status, and lung cancer stage. The assumption of proportional hazards was tested by including interaction terms between time and each explanatory variable; significant effects for these interactions indicate violation of the assumption. Where this occurred, the interaction terms were adjusted for in the final model.^16^ Since detectability of lesions may be associated with size and stage, which would be expected to progress over time, an additional analysis was conducted comparing stage at diagnosis and survival between cases diagnosed earlier and later than 6 weeks following initial CXR. This was intended to facilitate comparison of cancers that were diagnosed within 6 weeks despite a negative CXR result with those that were diagnosed later than 6 weeks.

## RESULTS

A total of 4698 patients were diagnosed with lung cancer, including 2129 (45.3%) with at least one GP-requested CXR in the year before diagnosis (Table 1). The study population included 113 (5.3%) patients who attended a service that allowed them to request their own CXR; the characteristics of that subpopulation have been described previously (see Supplementary Appendix S1 for details).^6^ The sensitivity of CXR, based on initial CXR (code 1 or 2), was 82.3% (95% confidence interval [CI] = 80.6% to 84.1%). A total of 370 (17.4%) patients had an initial CXR result that advised non-urgent further review or investigation (code 2). Of these patients, 191 (51.6%) had a second GP-requested CXR. The median duration to second CXR was 42 days (interquartile range [IQR] 28–57) and the result was negative in 19 cases (9.9%, 95% CI = 6.4% to 13.5%) (data not shown).

**Table 1.** Study population by initial chest X-ray group

**Variable**	**Initial CXR code 1**	**Initial CXR code 2**	**Initial CXR code 3**	**Initial CXR code 4**	**‘Positive’ (code 1 or 2)**	**‘Negative’ (code 3 or 4)**	**Total**
***n* (%)^a^**	1383 (65.0)	370 (17.4)	230 (10.8)	146 (6.9)	1753 (82.3)	376 (17.6)	2129

**Age, years, mean**	71	72	75	70	71	73	72

**Sex, male,** ***n* (%)**	753 (54.4)	189 (51.1)	121 (52.6)	72 (49.3)	942 (53.7)	193 (51.3)	1135 (53.3)

**CXR to diagnosis, median days (IQR)**	36 (23–63)	93 (55–154)	211 (181–296)	193 (87–279)	43 (27–78)	204 (105–287)	51 (29–107)

**Survival from CXR, median days (IQR)**	313 (126–877)	400 (163–964)	408 (238–958)	420 (214–1117)	328 (135–899)	412 (225–1011)	345 (148–920)

**Stage**							
I/II, *n* (%), (95% CI)	397 (28.7), (26.4 to 31.2)	111 (30.0), (25.4 to 35.0)	83 (36.1), (30.0 to 42.7)	43 (29.5), (22.4 to 37.7)	508 (29.0), (26.9 to 31.2)	126 (33.5), (28.8 to 38.6)	634 (29.8), (27.9 to 31.8)
III/IV, *n* (%), (95% CI)	981 (70.9), (68.4 to 73.3)	259 (70.0), (65.0 to 74.5)	147 (63.9), (57.3 to 70.1)	103 (70.5), (62.4 to 77.7)	1240 (70.7), (68.5 to 72.9)	250 (66.5), (61.4 to 71.2)	1490 (70.0), (68.0 to 71.9)
Unknown, *n* (%)	5 (0.4)	0	0	0	5 (0.3)	0	5 (0.2)

**Histology,** ***n* (%)**							
Small-cell	170 (12.3)	39 (10.5)	30 (13.0)	25 (17.1)	209 (11.9)	55 (14.6)	264 (12.4)
Non-small-cell	961 (69.5)	257 (69.5)	123 (53.5)	87 (60.0)	1218 (69.5)	210 (55.9)	1428 (67.1)
Other histologies^b^	—	—	—	—	12 (0.7)	5 (1.3)	17 (0.8)
Unknown	244 (17.6)	70 (18.9)	76 (33.0)	30 (20.5)	314 (17.9)	106 (28.2)	420 (19.3)

a *Percentages in some cases exceed 100 because of rounding.*

b *In order to maintain anonymity, numbers for CXR groups 1–4 have not been reported. CI = confidence interval. CXR = chest X-ray. IQR = interquartile range.*

A total of 324 patients (15.2%) had ≥2 CXRs before diagnosis (code 1–4), with sensitivity of these follow-up CXRs increasing only slightly from 82.3% (95% CI = 80.6% to 84.1%) on initial CXR to 83.6% (95% CI = 79.2% to 88.0%) on the subsequent CXR (Table 2, CIs not shown). Of the 376 patients who had an initial CXR that was negative (Table 1), 98 (26.1%) had at least one further CXR (Table 2).

**Table 2.** Number of GP-requested chest X-rays in year before diagnosis

**CXRs performed, *n***	**Patients, *n***	**Male, *n* (%)**	**Mean age, years**	**Positive CXR, *n* (%)**	**Previous CXR positive, *n* (%)**	**Stage I or II at diagnosis, *n* (%)**	**Median days from previous CXR (IQR)**	**Median days to diagnosis from initial CXR (IQR)**
**1**	1805	978 (54.2)	72	1527 (84.6)	—	523 (29.0)	—	44 (27–84)
**2**	277	126 (45.5)	72	244 (88.1)	185 (66.8)	83 (30.0)	49 (29–139)	128 (79–223)
**3**	43	21 (48.8)	70	37 (86.0)	26 (60.5)	13 (30.2)	74 (44–141)	239 (186–283)
**4**	4	^a^	^a^	4 (100.0)	3 (75.0)	^a^	96 (39–170)	340 (54–363)
**1, 2, 3, or 4** ^b^	2129	1135 (53.3)	72	1753 (82.3)	—	634 (29.8)	—	51 (29–107)
**2, 3, or 4** ^b^	324	156 (48.1)	72	271 (83.6)^c^	226 (69.8)^d^	111 (34.3)	49 (29–134)	148 (84–251)
**3 or 4** ^b^	47	23 (48.9)	70	40 (85.1)	28 (59.6)	14 (29.8)	67 (42–144)	251 (114–304)

a *Demographic data have been excluded to maintain patient anonymity.*

b *CXR results pertain to the first CXR in each row, not to the total of all CXRs, for example, for ‘1, 2, 3, or 4’ indicates that the first CXR was positive for 1753; row ‘2, 3, or 4’ indicates that the second CXR was positive in 271 out of 324 patients who had at least two CXRs.*

c *In those who had a negative initial CXR and who had a second CXR (n = 98), the second CXR code was 1 for 52 (53.1%), 2 for 16 (16.3%), 3 for 21 (21.4%), and 4 for 9 (9.2%).*

d *Of those who had two or more CXRs in the year before diagnosis, the initial CXR code was 1 for 35 patients (10.8%), 2 for 191 (59.0%), 3 for 53 (16.4%), and 4 for 45 (13.9%). CXR = chest X-ray. IQR = interquartile range.*

Median time from initial CXR to diagnosis for those with a ‘positive’ result was 43 days (IQR 27–78) compared with 204 days (IQR 105–287) for those who had a ‘negative’ CXR (Table 1). Further details of CXR results, median durations to diagnosis, and stage at diagnosis by group are displayed in Table 1 (see Supplementary Figures S1–S3 for details of Kaplan–Meier and Cox regression survival analyses for duration to diagnosis and CXR result).

Stage at diagnosis was similar across groups, with 634 (29.8%) patients diagnosed at stage I or II, including 508 (29.0%) who had a ‘positive’ initial CXR and 126 (33.5%) who had a ‘negative’ initial CXR (Table 1). There was no evidence of a statistically significant association between CXR result and stage at diagnosis, χ^2^ (1, *N* = 2124) 2.92, *P* = 0.09.

Patients who were diagnosed within 6 weeks of initial CXR regardless of CXR result were more likely to have stage III or IV disease (*n* = 775/880, 88.1% versus *n* = 715/1244, 57.5%, *P*<0.001) (Table 3) and small cell histology (*n* = 115/884, 13.0% versus *n* = 109/1245, 8.8%, *P*<0.001) (see Supplementary Table S1 for details). This suggests that late-stage disease and histology associated with rapidly progressive disease is more likely to be diagnosed rapidly, which could be due to the severity of presenting symptoms and/or more clear-cut radiological evidence of cancer. Among patients diagnosed ≥6 weeks (42 days) after initial CXR, there was evidence that those for whom the initial CXR was negative were more likely to have stage III or IV disease than those for whom the initial CXR was positive (*n* = 225/350, 64.3% versus *n* = 490/894, 54.8%, *P* = 0.002) (Table 4). Few patients with initial negative CXRs received a diagnosis of lung cancer within 6 weeks of initial CXR (*n* = 26/376, 6.9%) (Table 5). Of those who did have negative initial CXRs and were diagnosed within 6 weeks, almost all had stage III or IV disease (*n* = 25/26, 96.2%) (Table 6).

**Table 3.** Lung cancer stage with respect to diagnosis with lung cancer within or after 6 weeks (42 days) following initial chest X-ray^a^

	**Diagnosed within 6 weeks of initial CXR, *n* (%)**	**Diagnosed after 6 weeks of initial CXR, *n* (%)**
**Stage I/II**	105 (11.9)	529 (42.5)
**Stage III/IV**	775 (88.1)	715 (57.5)
**Total**	880	1244

a *Unknown stage excluded to maintain anonymity. Pearson’s χ^2^ demonstrated a statistically significant association between both late stage and diagnosis within 6 weeks, χ^2^ (1, N = 2124) 230.36, P*<*0.001. CXR = chest X-ray.*

**Table 4.** Lung cancer stage at diagnosis and initial chest X-ray results for those who were diagnosed after 6 weeks (42 days) following initial chest X-ray^a^

	**Patients diagnosed after 6 weeks of initial CXR, *n* (%)**	**Positive initial CXR, *n* (%)**	**Negative initial CXR, *n* (%)**
**Stage I/II**	529 (42.5)	404 (45.2)	125 (35.7)
**Stage III/IV**	715 (57.5)	490 (54.8)	225 (64.3)
**Total**	1244	894	350

a *Those with unknown stage are not included in order to maintain anonymity. Pearson’s χ^2^ test did demonstrate a statistically significant association, χ^2^ (1, N = 1244) 9.24, P = 0.002. CXR = chest X-ray.*

**Table 5.** Result of initial chest X-ray and diagnosis within or after 6 weeks (42 days)^a^

	**Diagnosed within 6 weeks of initial CXR, *n* (%)**	**Diagnosed after 6 weeks of initial CXR, *n* (%)**
**CXR positive**	858 (97.1)	895 (71.9)
**CXR negative**	26 (2.9)	350 (28.1)
**Total**	884	1245

a *Pearson’s χ^2^ test demonstrated a statistically significant association between positive CXR and diagnosis within 42 days, χ^2^ (1, N = 2129) 225.24, P*<*0.001. CXR = chest X-ray.*

**Table 6.** Stage and initial chest X-ray results for those who were diagnosed within 42 days (6 weeks)^a^

	**Patients diagnosed *within* 6 weeks of initial CXR, *n* (%)**	**Positive initial CXR, *n* (%)**	**Negative initial CXR, *n* (%)**
**Stage I/II**	105 (11.9)	105 (12.3)	1 (3.8)
**Stage III/IV**	775 (88.1)	749 (87.7)	25 (96.2)
**Total**	880	854	26

a *Pearson’s χ^2^ test did not demonstrated a statistically significant association between stage and CXR result, χ^2^ (1, N = 880) 1.67, P = 0.196. The result is not significant at P*<*0.05, 1 degree of freedom. Patients for whom stage was unknown are not included in order to maintain anonymity. CXR = chest X-ray.*

Survival analysis demonstrated no adverse effect on survival for those with a negative CXR result compared with those with a positive CXR. Adjustment for covariates using Cox proportional hazards regression found that those with positive CXR results had poorer survival relative to the negative CXR group (hazard ratio 1.35, 95% CI = 1.19 to 1.52, *P*<0.001) (see Supplementary Figure S1 for details).

## DISCUSSION

### Summary

This study estimates that the sensitivity of CXR for lung cancer diagnosed within 1 year among patients presenting to primary care is 82.3% (95% CI = 80.6% to 84.1%). Of the patients who had a CXR in the year before their diagnosis with lung cancer, those with positive results had a median duration to diagnosis of 43 days compared with 204 days for those with a negative initial CXR.

However, the study did not find evidence of a direct association between failure to detect lung cancer on CXR and adverse stage at diagnosis or survival. It is possible that such associations do exist but are obscured by confounding as a result of the retrospective observational study design or because the study lacked the statistical power to detect such associations.

### Strengths and limitations

To the authors’ knowledge, this study is the first to analyse CXR results systematically with respect to time to diagnosis, stage at diagnosis, and survival. It also draws on by far the largest published population in estimating the sensitivity of CXR for lung cancer in symptomatic patients, exceeding by more than five-fold the total population of three studies of low bias identified in a 2019 systematic review (*n* = 380).^5^ The classification of positive and negative results is poorly defined in many of the studies that have previously reported the sensitivity of CXR. The present study employed a systematic approach to classifying CXR results, which was validated and refined using a sample of CXR results before the study began.

Smoking status, comorbidities, and the symptoms that prompted investigation with CXR were not available. It is not possible to know whether CXRs were requested because of respiratory symptoms or symptoms stipulated in guidance from the National Institute for Health and Care Excellence.^17^ However, this reflects real-world clinical practice, and investigations that lead to a lung cancer detection may be initiated without malignancy having been initially considered as a likely diagnosis.

The study population was drawn from a single city; therefore, it is possible that local patterns of demography or clinical practice may mean the findings are less applicable to other settings. However, Leeds is broadly representative of the wider English population in terms of age, ethnicity, and deprivation.^18^

A period of 1 year from CXR to diagnosis was chosen to determine sensitivity, reflecting much of the existing literature.^5^ One year is a period in which it would be likely that a macroscopic lesion would be present. The choice of time period has important consequences for sensitivity because choosing a longer period, such as 2 years, would likely result in lower sensitivity, while a shorter period, such as 6 months, would probably lead to higher sensitivity. Estimates derived from screening studies suggest that, in a large proportion of cases, lung cancer develops over years before detection, although a small proportion of cancers develop more rapidly.^15^^,^^19^^–^^21^ It is possible that, in some cases, the lung cancer did not constitute a macroscopic lesion at the time at which the initial CXR was performed.

Because of the retrospective observational design of this study, no definitive conclusions can be drawn from the lack of observed association between detection of lung cancer and stage at diagnosis or survival. It is likely that the detectability of lung cancers has an independent relationship with stage and survival. Larger tumours may have been more detectable and could also have been more likely to represent late-stage disease. Lesions that were initially not detected could, however, have been more likely to be faster growing tumours, with poorer prognoses, akin to ‘interval cancers’ described in screening studies.^22^ Exploratory analyses in this study suggest that late-stage disease is associated with diagnosis within 6 weeks. Since the current study did not find evidence that this effect is mediated by CXR result, it is possible that patients with more advanced disease are more likely to be diagnosed early. While this may support the so-called ‘sick quick’ theory, it is important to acknowledge that such observations in this context are speculative.^23^

### Comparison with existing literature

A 2019 systematic review for the sensitivity of CXR for lung cancer in symptomatic patients identified three studies with estimates of 79% (95% CI = 68% to 91%), 77% (95% CI = 65% to 84%), and 80% (95% CI = 73% to 87%).^5^ Sensitivity in the present study (82.3%) was consistent with previous estimates, although the larger sample size has yielded tighter CIs (95% CI = 80.6% to 84.1%) than previous investigations. The sensitivity of a subset of patients who were represented in this study population has previously been published (75%, 95% CI = 68% to 83%).^6^ Sensitivity is affected by the prevalence of the disease and differences in the spectrum of disease, which might have contributed to the higher sensitivity in this study, since all of the patients in the present study had a diagnosis of lung cancer.^24^

In a Danish study, 12 patients with lung cancer who had a negative CXR result had a median duration from presentation to GP to diagnosis of 161 days compared with 27 days for those with a positive CXR.^7^ In another retrospective study, diagnosis was ‘missed’ on the CXRs of 14 patients who had experienced an additional median delay of 101 (IQR 48–339) days.^10^

The association between duration to diagnosis and survival is known to be complex. Tørring *et al* ^25^ and Redaniel *et al* ^26^ found increasing mortality with longer diagnostic intervals; however, they also observed higher mortality with short diagnostic intervals. A systematic review that examined time to diagnosis and outcomes for lung cancer presented *‘mixed findings’*, with similar numbers of studies demonstrating positive, negative, and no associations.^27^ Such observations are likely to be related to the clinical heterogeneity of cancer presentations. While undetected cancers will progress unchecked by treatment, rapidly progressive cancers that confer poor outcomes may also have shorter diagnostic intervals both through their more florid clinical presentation and shorter overall survival. In this study, it is possible that any adverse consequences of failure to detect cancer have been obscured by comparison with cancers that were more advanced and therefore more likely to be detected on CXR.

The present study found that 45.3% of patients diagnosed with lung cancer had a GP-requested CXR in the year before diagnosis, which is broadly similar to that found in a larger study,^28^^,^^29^ but less than that found in an older cohort of 247 patients (66%).^30^ In England, it is estimated that 48% of lung cancer diagnoses result from GP referrals, although it is not known how many of these referrals occurred following a GP-requested CXR.^31^

### Implications for research and practice

This study suggests that CXR fails to identify lung cancer in around 17.7% of patients with the disease in the year before diagnosis. Therefore, GPs should be mindful that a negative CXR does not necessarily exclude lung cancer. It is also important for GPs to recognise that, although the risk of lung cancer with a negative CXR for most symptoms is low, the risk for patients with unexplained haemoptysis is almost 3% and urgent referral for suspected cancer is often warranted for this symptom, regardless of CXR result.^6^^,^^17^

Compared with many similar countries, the UK has less capacity for more advanced imaging modalities such as computed tomography (CT).^32^ In the UK several local initiatives have expanded access to CT for GPs in recent years in order to help expedite cancer diagnoses, while improving radiology capacity nationwide has been recognised as a policy priority.^33^^,^^34^ Given both the deficit in 2-week referrals for suspected lung cancer and the backlog in CT imaging as a result of the COVID-19 pandemic, making effective use of CXR capacity is likely to remain crucial in optimising lung cancer diagnosis in the coming years.^35^^–^^37^ For GPs, recognising those patients who may warrant additional investigation or referral despite unremarkable CXR will remain a challenge. In this context, a prospective study that compares CXR with CT in symptomatic patients with careful consideration of the benefits, harms, and health–economic implications may be required to understand whether transitioning to CT as the first-line investigation would be justified.

In this study, for the 15.2% of patients who had a further CXR in the year before diagnosis, sensitivity increased only slightly from 82.3% on the initial CXR to 83.6% on the repeat CXR. Meanwhile, in 9.9% of those who had another CXR following a result that indicated non-urgent followup, this result was negative. Therefore, even for patients who have a repeat CXR that is negative, GPs should not dismiss the possibility of lung cancer if symptoms persist. In such circumstances further actions could include reassessment after a suitable interval, requesting imaging with another modality such as CT, or asking for advice from colleagues in respiratory medicine.

The finding that patients who had a positive CXR with a recommendation for non-urgent follow-up had a median duration to diagnosis almost three times longer than those who have a positive CXR and a recommendation for urgent further investigation suggests that efforts to expedite diagnosis for this group of patients may be warranted. It is also striking that only about half of those who had a CXR recommending non-urgent follow-up actually had a further GP-requested CXR in the year before diagnosis. As this study recorded only GP-requested CXRs, it is possible that appropriate management was instituted, for example, through referral to secondary care, but further audit or quality improvement work would be required to understand whether the diagnosis for these patients could have been expedited.
